# Prognostic significance and regulatory role of ACOT7 in the tumor immune microenvironment of breast invasive ductal carcinoma: a multi-omics analysis

**DOI:** 10.3389/fimmu.2026.1735908

**Published:** 2026-02-25

**Authors:** Chonghui Song, Yinglan Quan, Yuxin Shan, Yantong Chen, Juan Du, Kunwei Li, Ning Li

**Affiliations:** 1 Faculty of Life Science and Techonology & The Affiliated Anning First People’s Hospital, Kunming University of Science and Technology, Kunming, China; 2 Faculty of Life Science and Techonology, Kunming University of Science and Technology, Kunming, China; 3 Imaging Department, Yunnan Maternal And Child Health Hospital, Kunming, China

**Keywords:** ACOT7, bioinformatics analysis, biomarker, breast invasive ductal carcinoma, immune infiltration

## Abstract

**Background:**

Invasive Ductal Carcinoma (IDC), which is the most common histological subtype of breast cancer, is highly aggressive and progresses rapidly. Acyl-CoA thioesterase 7 (ACOT7) is a key regulator of cell survival, the cell cycle, and lipid and glucose metabolism. However, the mechanism of ACOT7 in IDC is still unclear. Our study aims to investigate the clinical significance of ACOT7 in IDC.

**Methods:**

A comparative analysis of ACOT7 expression in IDC and matched normal tissues was performed using the limma R package on datasets from GEO and TCGA. Prognostic evaluation was conducted using Kaplan-Meier survival curves from the Kaplan-Meier plotter. Furthermore, a protein-protein interaction (PPI) network of ACOT7 was constructed by GeneMANIA, and the correlation between ACOT7 expression and the level of tumor immune infiltration was explored via the TIMER database. In order to further analyze the biological functions of ACOT7, we performed Gene Ontology (GO) analysis, Kyoto Encyclopedia of Genes and Genomes (KEGG) pathway enrichment analysis, and Gene Set Enrichment Analysis (GSEA) using the clusterProfiler package. To validate this, we profiled ACOT7 mRNA expression across clinical samples (tumor and adjacent normal) and *in vitro* models (cell lines) via Real-Time quantitative Polymerase Chain Reaction (RT-qPCR).

**Results:**

Bioinformatic analysis of public databases revealed that ACOT7 mRNA expression was significantly upregulated in IDC patients compared to normal tissues. Elevated ACOT7 expression was associated with poorer overall survival, a finding further validated in cell lines and clinical tissue samples. Furthermore, ACOT7 transcriptional levels showed a significant correlation with the degree of tumor immune infiltration. Functional enrichment analysis indicated that ACOT7 is primarily involved in cancer-related regulation, autoimmune diseases, and multiple metabolic pathways.

**Conclusion:**

Our study indicates that elevated ACOT7 expression is a significant marker of adverse clinical outcomes. This effect it likely mediated through the remodeling of the tumor immune microenvironment and the reprogramming of metabolic pathways, which collectively fuel the malignant procession of IDC. These results provide a solid theoretical foundation for targeting ACOT7 as both a prognostic biomarker and a potential therapeutic target in IDC.

## 1 Introduction

Despite its high incidence, breast cancer continues to represent a leading cause of cancer-related mortality in the female population globally. Although the progress of early screening and comprehensive treatment has significantly improved the prognosis of patients, the overall incidence rate is still on the rise (1, 2). Among the various subtypes of breast cancer, invasive ductal carcinoma (IDC) is the most prevalent, accounting for approximately 47%-79% of all cases. This subtype is highly aggressive and progresses rapidly (3, 4). Particularly in its advanced stages, IDC is associated with poor treatment efficacy and low patient survival rates, posing a major clinical challenges (5). In recent years, the development and integration of multi-omics technologies (such as genomics, transcriptomics, etc.) have provided powerful new tools for the in-depth analysis of the tumor immune microenvironment and immunoregulatory mechanisms in breast cancer (6). Therefore, utilizing multi-omics databases to systematically identify key molecular events related to immune regulation in IDC and to construct a comprehensive prognostic biomarker framework has become a major research focus (7, 8).

The ACOT7 gene encodes Acyl-CoA thioesterase 7, is a cytosolic metabolic enzyme that primarily catalyzes the hydrolysis of long-chain acyl-CoA into free fatty acids and coenzyme A, thereby precisely regulating intracellular lipid metabolic homeostasis. Evidence from the literature underscores the pivotal role of ACOT7 in coordinating cell survival (9), cell cycle progression (10), immune cell infiltration (11), as well as lipid and glucose metabolism (12). However, the specific mechanism of this enzyme in the development of IDC is still unclear. Given that lipid metabolic reprogramming is a central hallmark of cancer, in-depth exploration of how ACOT7 drives IDC’s malignant progression (including proliferation, invasion, and therapy resistance) by influencing fatty acid metabolism, energy supply, or signal transduction will help uncover novel metabolic vulnerabilities and provide a crucial basis for developing potential targeted strategies.

This study will analyze the expression differences of ACOT7 in breast invasive carcinoma (BRCA) tissues and adjacent tissues, and clarify its clinical significance based on GEO and TCGA databases, and the relevant results will be further validated by Real-Time quantitative Polymerase Chain Reaction (RT-qPCR). Furthermore, we will further explore the potential function of ACOT7 in BRCA by establishing PPI network, combined with immune infiltration and pathway enrichment analyses. Based on this integrated approach, we aim to achieve a deeper understanding of the mechanistic role of ACOT7 in BRCA, thereby providing novel insights for assessment in BRCA patients.

## 2 Materials and methods

### 2.1 Analysis based on the GEO database

In this study, two microarray datasets (GSE21422 and GSE29044) were retrieved from the NCBI Gene Expression Omnibus (GEO, https://www.ncbi.nlm.nih.gov/geo). The combined cohort comprised 119 human samples, consisting of 78 IDC tissues and 41 adjacent normal tissues (detailed in **Table 1**). Differential expression of ACOT7 between tumor and normal tissues was analyzed using the limma R package, with significance thresholds set at |log2 Fold Change (FC)| > 0.5 and an adjusted p-value < 0.01. Visualization was performed using volcano plots (ggplot2) and heatmaps (pheatmap).

**Table 1:** Dataset information of invasive ductal carcinoma of the breast.

Dataset	Normal (cases)	Tumor (cases)	Sequencing platform	Experiment type	Organism
GSE21422	5	5	GPL570	Expression profiling by array	Homo sapiens
GSE29044	36	73	GPL570	Expression profiling by array	Homo sapiens

### 2.2 Analysis based on the TCGA database

Transcriptomic data and corresponding clinical information for breast invasive carcinoma (BRCA) were retrieved from The Cancer Genome Atlas (TCGA) via the Genomic Data Commons (GDC) Data Portal (https://cancergenome.nih.gov). Following the exclusion of non-invasive ductal carcinoma cases based on pathological records, a total of 793 breast invasive carcinoma samples and 93 normal samples were included. The limma R package was utilized for data preprocessing (log2 transformation and quantile normalization) and to identify differentially expressed genes (DEGs). DEGs were filtered using the criteria: |log2 Fold Change (FC)| > 0.5; adjusted p-value < 0.01.The results visualized using volcano plots (ggplot2) and heatmaps (pheatmap).

### 2.3 Human protein atlas

The Human Protein Atlas (HPA, https://www.proteinatlas.org) is a comprehensive database mapping protein localization and expression in human tissues via immunohistochemistry (IHC). To assess the tissue distribution of ACOT7, we retrieved IHC staining images from the HPA for both invasive breast carcinoma and normal breast tissues. Specifically, ACOT7 protein expression was evaluated in invasive breast carcinoma tissue from a 43-year-old female and normal breast tissue from a 45-year-old female using antibody HPA025762.

### 2.4 Kaplan-meier plotter

Kaplan-Meier plotter (https://kmplot.com) is an online tool designed to assess survival outcomes for various cancers based on gene expression data. It integrates extensive transcriptomic and clinical survival data from public databases (e. g., GEO, TCGA), enabling researchers to quickly assess the association between the expression of specific genes and patient survival. We utilized this tool to evaluate the prognostic value of ACOT7. Specifically, we analyzed the association between ACOT7 expression and Overall Survival (OS), Relapse-Free Survival (RFS), and Distant Metastasis-Free Survival (DMFS) in patients with breast cancer.

### 2.5 Screening and network construction of ACOT7 interacting proteins

The Gene Multiple Association Network Integration Algorithm (GeneMANIA; http://www.genemania.org) is a web-based interface used for predicting gene functions via the integration of diverse genomic and proteomic datasets. In this study, GeneMANIA was employed to construct a functional interaction network centered on ACOT7.

### 2.6 Immune cell infiltration analysis of ACOT7

The Tumor Immune Evaluation Resource (TIMER, https://cistrome.shinyapps.io/timer/) is a comprehensive web platform for analyzing the interplay between gene expression and immune cell infiltration in the tumor microenvironment. It integrates genomic and transcriptomic data from 32 cancer types in TCGA. In this study, we utilized the ‘Gene’ and ‘SCNA’ (Somatic Copy Number Alteration) modules of TIMER to assess the association between ACOT7 expression, as well as its copy number status, and immune cell infiltration levels in the TCGA BRCA cohort.

### 2.7 Functional enrichment analysis

Genes significantly associated with ACOT7 expression (both positive and negative) were identified from the TCGA BRCA dataset. Gene Ontology (GO) functional annotation (including Biological Process [BP], Cellular Component [CC], Molecular Function [MF]) and Kyoto Encyclopedia of Genes and Genomes (KEGG) pathway enrichment analyses were performed using the clusterProfiler R package. Enriched terms with a p-value < 0.05 were considered statistically significant, and the results were visualized by ggplot2.

### 2.8 Gene set enrichment analysis

To elucidate the biological mechanisms associated with ACOT7, Gene Set Enrichment Analysis (GSEA) was performed. First, a ranked gene list was generated based on the correlation between ACOT7 expression and the whole transcriptome. The KEGG gene set collection (c2.cp.kegg.v7.2.symbols.gmt) was utilized as the reference database using the clusterProfiler package. Statistical significance was assessed using a default permutation test (1000 permutations). Gene set were considered significantly enriched using the following criteria: Normalized Enashment Score (NES) > 1.0, nominal p-value < 0.05 and False Discovery Rate (FDR) < 0.25.

### 2.9 Collection of clinical specimens

This study was approved by the Ethics Committee of Anning First People’s Hospital (Approval No.: 2025-007-01). Tumor tissues and paired adjacent non-tumor tissues were collected from 15 patients with histologically confirmed invasive carcinoma. Following surgical resection, all specimens were immediately flash-frozen in liquid nitrogen and stored at -80 °C. Patients who had received neoadjuvant therapy were excluded from the study.

### 2.10 Cell culture

Three breast invasive carcinoma cell lines (T47D, BT549 and MCF7) and a normal breast epithelial cell line (MCF10A) were obtained from Beina Biological (China). T47D cells were cultured in high-glucose DMEM (Gibco); BT549 cells were maintained in RPMI 1640 medium (Gibco) supplemented with Insulin-Transferrin-Selenium (100 × ITS, YEASEN, China); MCF7 cells were grown in high-glucose DMEM containing 1% Non-Essential Amino Acid (NEAA, Procell, China) and 10 µg/mL Insulin (Procell, China); and MCF10A cells were cultured in specialized MCF10A medium. All complete medium were supplemented with 10% fetal bovine serum (FBS, Gibco) and 1% antibiotic-antimycotic solution (containing 10,000 U/mL penicillin, 10 mg/mL streptomycin, and 25 µg/mL amphotericin B; Servicebio, China). All cell lines were incubated at 37 °C in a humidified atmosphere containing 5% CO_2_.

### 2.11 RNA extraction and real-time quantitative polymerase chain reaction

Total RNA was extracted from cell lines and clinical tissues using Trizol reagent, following the manufacturer’s instructions. Subsequently, RNA was reverse transcribed into cDNA. The cDNA of the indicated gene was quantified using the SYBR Master mixture (YEASEN, China) on the Xi’an Tian-long automatic medical PCR system. The thermal cycling conditions consisted of an initial denaturation at 95 °C for 2 min, followed by 40 cycles, each cycle including denaturation at 95 °C for 10 s; annealing/extension at 60 °C for 30 s, and finally incubation at 72 °C for 5 min. The relative mRNA expression levels were calculated using the 2-ΔΔCt method, normalized to the internal reference gene, GAPDH. All primer sequences were synthesized by Sangon Biotech (Shanghai, China) and are listed in **Table 2**.

**Table 2:** RT-qPCR primer sequences.

Primer	5′→3′
ACOT7-F	CACGGAGGTGTGACCATGAA
ACOT7-R	ACGGAAGCTGTGACGATGTT
GAPDH-F	CAGGAGGCATTGCTGATGAT
GAPDH-R	GAAGGTGGCGGCTCATTT

RT-qPCR, Real-Time quantitative Polymerase Chain Reaction; ACOT7, Acyl-CoA thioesterase 7; GAPDH, Glyceraldehyde-3-Phosphate Dehydrogenase.

### 2.12 Statistical analysis

Statistical analyses were performed using GraphPad Prism 10.1.2. Quantitative data are presented as the mean ± standard deviation (SD) from three independent experiments. Comparisons between two groups were analyzed using Student’s t-test, while comparisons among multiple groups were assessed using one-way ANOVA. A p-value < 0.05 was considered statistically significant.

## 3 Results

### 3.1 Bioinformatic analysis of ACOT7 expression using the GEO and TCGA databases

**Figure 1:**
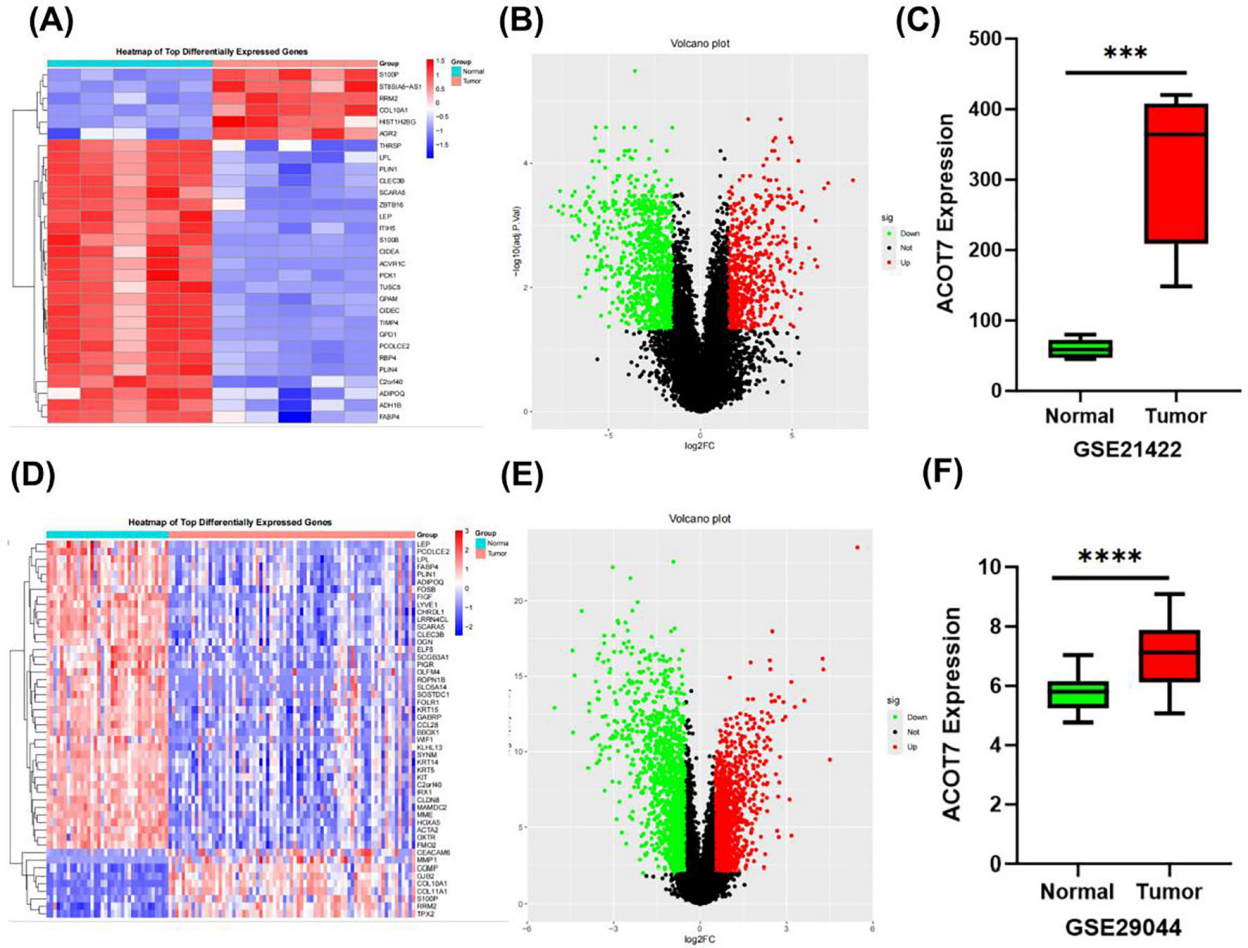
ACOT7 expression profile in invasive ductal carcinoma relative to normal breast tissues: an analysis of GEO datasets. **(A)** The heatmap represents a comparative analysis of gene expression across invasive ductal carcinoma and matched normal breast tissues from the GSE21422 dataset. **(B)** Differential analysis of the GSE21422 dataset identifies 1,471 genes with significant expression changes (611 upregulated and 860 downregulated), as visualized in the volcano plot. **(C)** Comparison of ACOT7 expression levels between invasive ductal carcinoma tissues and normal breast samples according to the GSE21422 dataset. **(D)** Heat map displaying differentially expressed genes in IDC tissues compared to normal tissues from GSE29044 dataset. **(E)** This volcano plot depicts the differential gene expression profile between invasive ductal carcinoma and normal breast tissues based on the GSE21422 dataset. **(F)** Comparison of ACOT7 expression levels between invasive ductal carcinoma tissues and normal breast samples according to the GSE29044 dataset. Triple asterisk (***) indicates P<0.001; quadruple asterisk (****) indicates P<0.0001. ACOT7: Acyl-CoA Thioesterase 7; IDC: Invasive Ductal Carcinoma.

**Figure 2:**
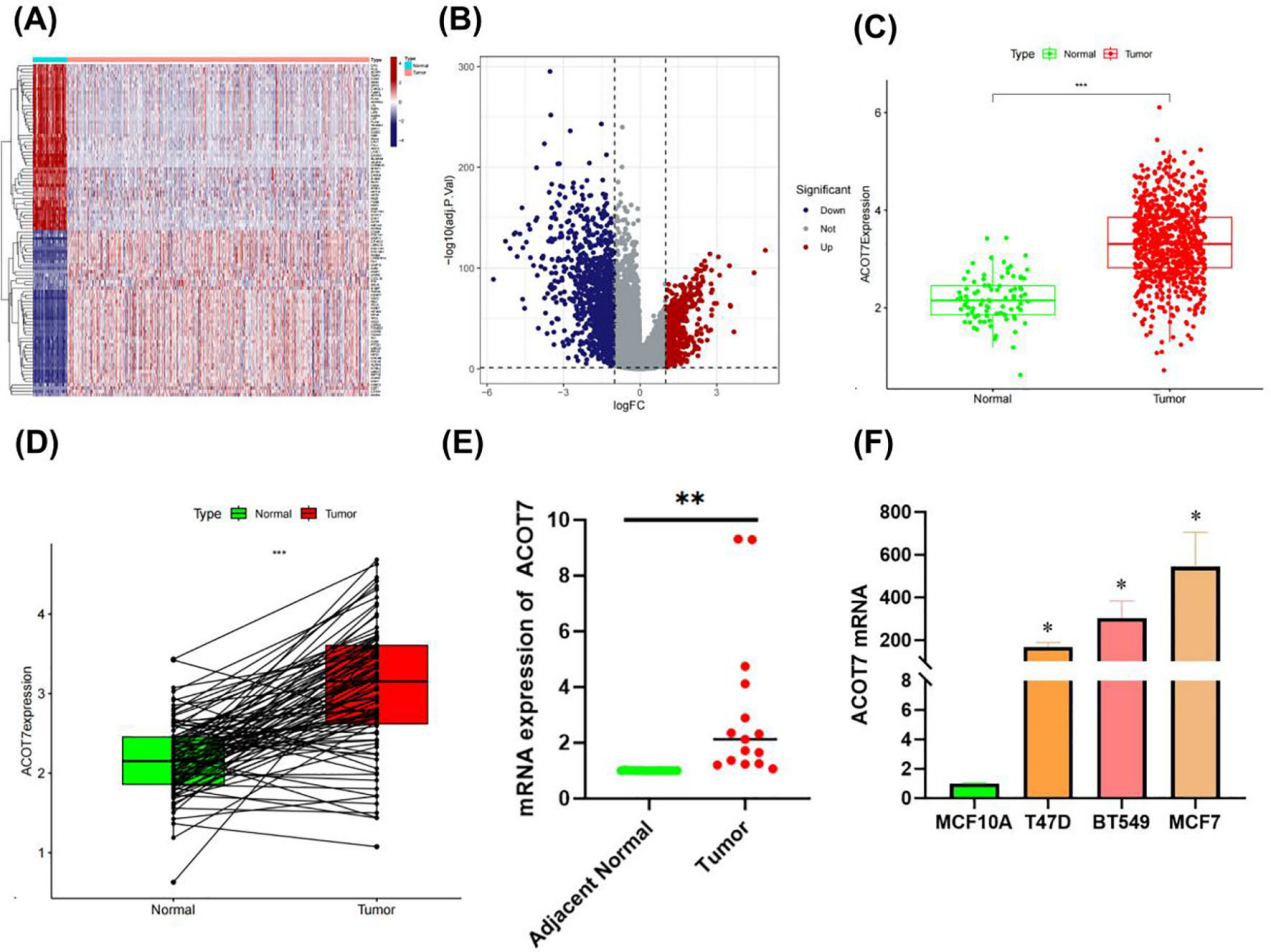
Profiling ACOT7 expression in invasive ductal carcinoma versus normal breast tissues leveraging TCGA data. **(A)** Heat map displaying expressed genes in TCGA BRCA. **(B)** Differential analysis of the TCGA-BRCA dataset, identifying 1,723 genes with significant expression changes (613 upregulated and 1,110 downregulated. **(C)** Analysis of ACOT7 Expression in BRCA versus Normal Breast Tissue from the TCGA Database. **(D)** Comparison of ACOT7 expression levels between normal breast samples and matched BRCA tissues based on TCGA data. **(E)** Quantification of ACOT7 mRNA expression in invasive breast carcinoma and adjacent non-tumor tissues by RT-qPCR. **(F)** mRNA expression levels of ACOT7 in breast cancer cell lines (T47D, BT549, MCF7) and normal human mammary epithelial cell line (MCF10A) detected by RT-qPCR. Asterisk (*) indicates P < 0.05; double asterisk (**) indicates P < 0.01; triple asterisk (***) indicates P < 0.001. ACOT7, Acyl-CoA Thioesterase 7; BRCA, Breast Invasive Carcinoma; RT-qPCR, Real-time quantitative Polymerase Chain Reaction.

Analysis of the two GEO databases consistently revealed a pronounced upregulation of ACOT7 transcripts in invasive ductal carcinoma relative to non-malignant controls (**Figures 1A–F**). Consistent results were obtained from differential expression analysis based on the TCGA database, which examined ACOT7 expression in BRCA (**Figures 2A–C**). Given the unequal number of tumor and normal samples in TCGA BRCA, the paired analysis was performed, revealing a significant increase in ACOT7 gene expression in breast tumor samples (**Figure 2D**). Furthermore, to explore the expression of ACOT7 in BRCA, we first analyzed clinical tissue samples. Analysis revealed a significant upregulation of ACOT7 mRNA in invasive breast cancer compared to adjacent normal tissues (P < 0.01; **Figure 2E**). Subsequently, we verified this trend at the cell line level. Compared to the normal mammary epithelial cell line MCF10A, ACOT7 expression was significantly elevated across a panel of breast cancer cell lines, including T47D, BT549, and MCF7 (P < 0.05; **Figure 2F**). Collectively, these results indicate that the upregulation of ACOT7 may be implicated in the pathogenesis and progression of breast cancer.

### 3.2 The protein expression of ACOT7 in BRCA tissues

**Figure 3:**
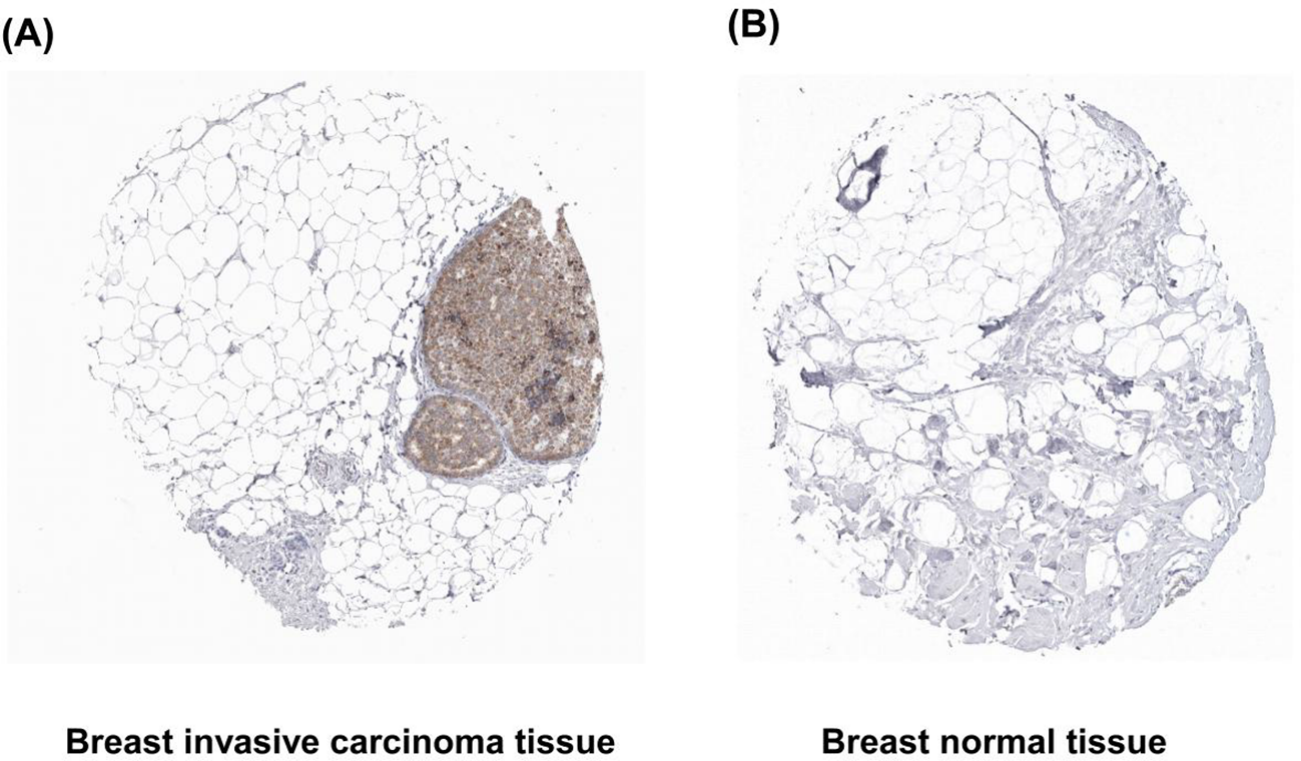
Representative immunohistochemical staining images of normal breast and invasive ductal carcinoma tissues from the HPA. **(A)** Invasive breast carcinoma tissue. **(B)** Normal breast tissue. The level of ACOT7 protein in invasive carcinoma tissues was increased. HPA, The Human Protein Atlas; ACOT7, Acyl-CoA Thioesterase 7.

We used HPA online database to analyze the protein expression of ACOT7 in BRCA tissues and normal breast tissues. The results revealed a significantly higher level of ACOT7 expression in BRCA tissue samples compared to normal breast tissue samples (**Figure 3**).

### 3.3 Survival analysis of the key gene ACOT7

**Figure 4:**
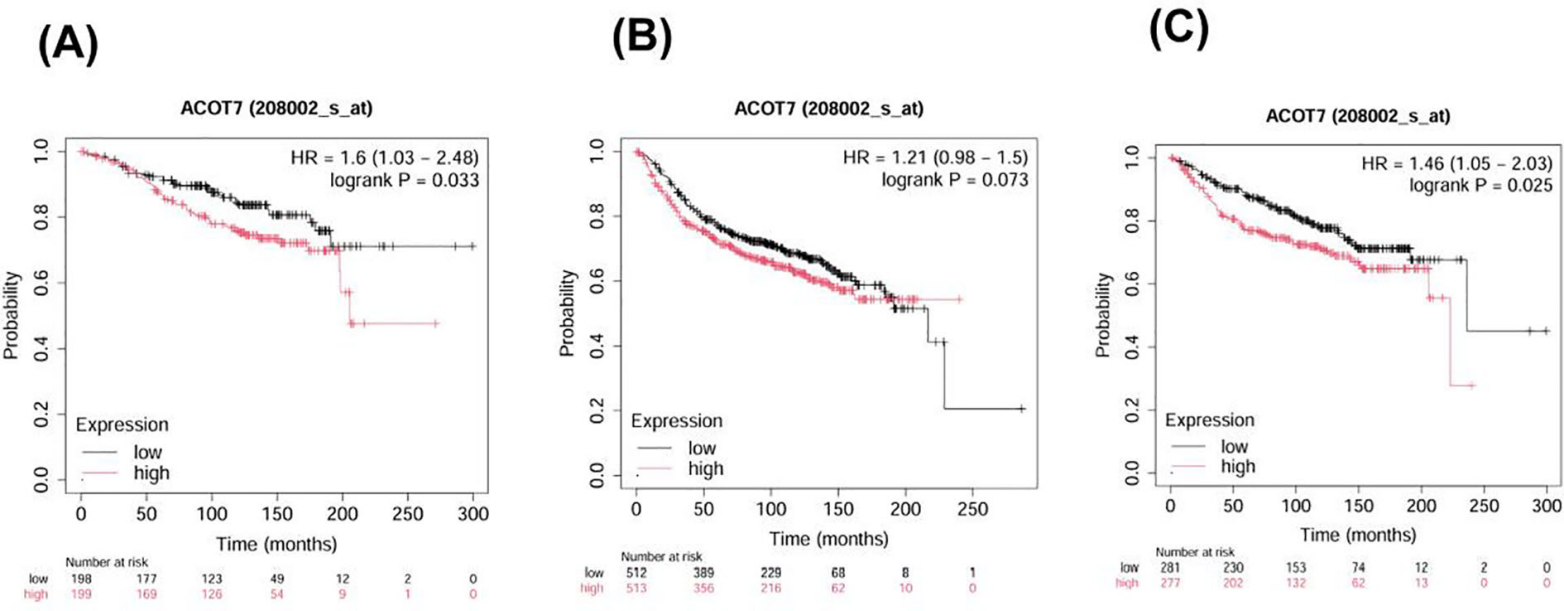
The effect of ACOT7 expression levels on the survival prognosis of BRCA patients. **(A)** The OS curve associated with ACOT7 expression in BRCA. **(B)** The RFS curve associated with ACOT7 expression in BRCA. **(C)** The DMFS curve associated with ACOT7 expression in BRCA. ACOT7, Acyl-CoA Thioesterase 7; BRCA, Breast Invasive Carcinoma; OS, Overall survival; RFS, Relapse-free survival; DMFS, Distant metastasis-free survival.

To evaluate the prognostic impact of ACOT7, we utilized the Kaplan-Meier plotter to stratify patients into high and low expression cohorts. Elevated ACOT7 levels were significantly associated with diminished overall survival and distant metastasis-free survival, identifying it as an adverse prognostic factor in BRCA (**Figures 4A–C**). No statistically significant difference was observed in relapse-free survival (P = 0.073 > 0.05).

### 3.4 Screening of ACOT7 interacting proteins and network construction results

**Figure 5:**
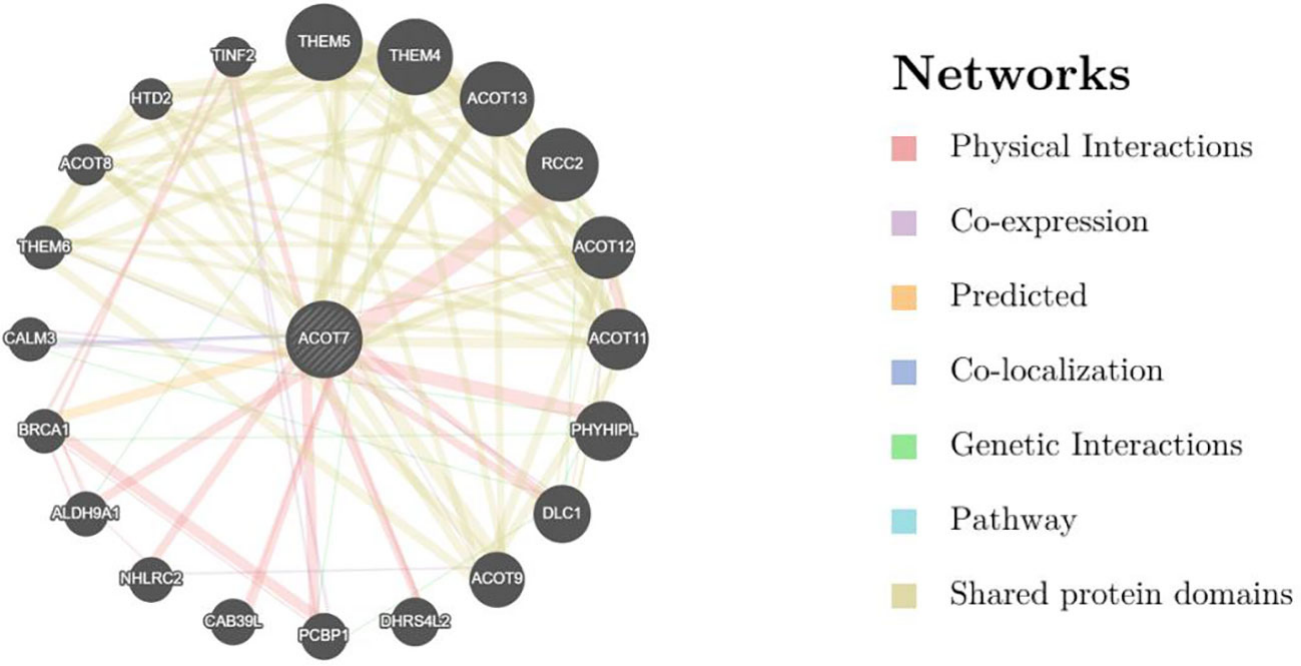
Screening and network construction of ACOT7 interacting proteins. Analysis of interactions between ACOT7 and other genes/proteins in BRCA patients using the GeneMANIA database. (Edge thickness represents interaction strength, colors denote interaction types, and node size corresponds to protein scores.) ACOT7, Acyl-CoA Thioesterase 7; BRCA, Breast Invasive Carcinoma.

GeneMANIA interaction network analysis of ACOT7 (**Figure 5**) showed that its core interacting proteins were significantly enriched in biological processes closely related to tumorigenesis and progression. In addition to multiple acyl-CoA thioesterase members (including ACOT11, ACOT12, ACOT13, THEM4, and THEM5) in the lipid metabolism pathway, which are closely related to ACOT7 through co-expression, several key cancer-related proteins were identified in the network. These included the tumor suppressor DLC1, DNA damage repair protein BRCA1, and mitotic regulator RCC2. These results suggest that ACOT7 is not only participates in the regulation of fatty acid metabolism, but also may influence cell cycle progression and genomic stability through metabolic reprogramming or non-metabolic functions, thereby contributing to tumorigenesis. In addition, the coexistence of other metabolism-related proteins such as ALDH9A1 and HTD2 broadens the scope of ACOT7’s potential functions within the tumor metabolic network, paving the way for further investigation into the molecular mechanisms of ACOT7 in cancer and other diseases.

### 3.5 ACOT7 is associated with immune infiltration of BRCA

**Figure 6:**
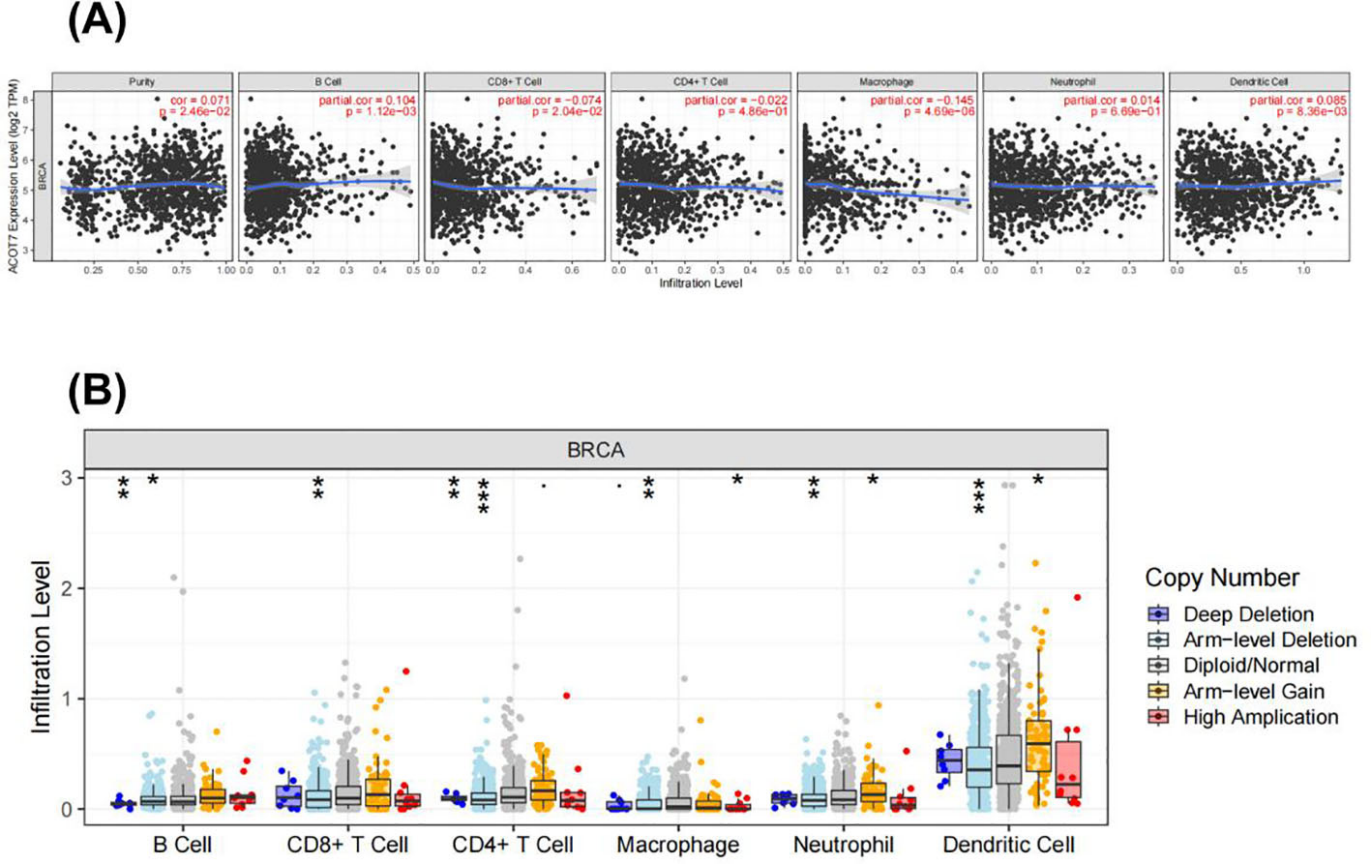
Association between ACOT7 expression and immune infiltration levels in breast cancer. (A) ACOT7 expression was positively correlated with tumor purity and significantly associated with increased infiltration of B cells and dendritic cells, while showing negative correlations with macrophages, CD4+ T cells, and CD8+ T cells. No significant association was observed with neutrophil infiltration. (B) The copy mutation of ACOT7 in BRCA may partially suppress the immune infiltration levels of macrophages and CD8+ T cells. Asterisk (*) indicates P< 0.05; double asterisk (**) indicates P<0.01; triple asterisk (***) indicates P<0.001. ACOT7: Acyl-CoA Thioesterase 7; BRCA: Breast Invasive Carcinoma.

We assessed the association of ACOT7 expression with immune cell infiltration levels in BRCA via the TIMER database. The results showed that ACOT7 expression was positively correlated with tumor purity (partial.cor = 0.071) and infiltration of B cells (partial.cor = 0.104) and dendritic cells (partial.cor = 0.085). On the contrary, it was negatively correlated with the infiltration of macrophages (partial.cor = -0.145) and CD8+ T cells (partial.cor = -0.074), while no significant association was observed with neutrophil infiltration (P = 0.669; **Figures 6A, B**). These data suggest that ACOT7 may play a dual role in the tumor immune microenvironment: on the one hand, it enhances immune surveillance by promoting the anti-tumor immune response of B cells and dendritic cells; on the other hand, it participates in the immune escape process by inhibiting the function of macrophages and CD8+ T cells. This effect may be related to its mechanism of regulating lipid metabolism and influencing macrophage M1/M2 polarization and inflammatory phenotype transformation.

### 3.6 The functional enrichment landscape of ACOT7 in breast cancer

**Figure 7:**
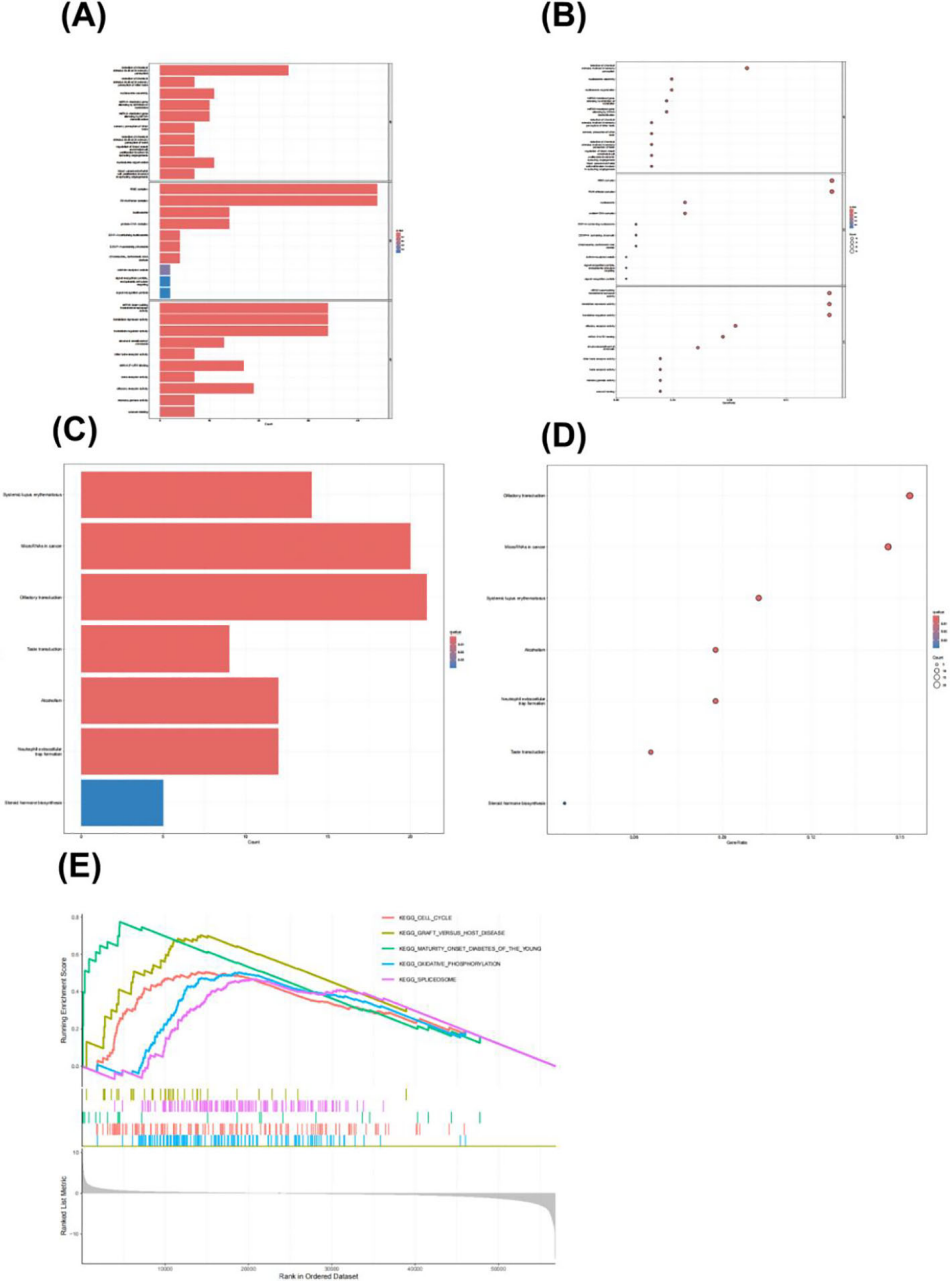
Functional enrichment results of ACOT7 in breast cancer (BRCA). **(A, B)** GO enrichment analysis of ACOT7 in BRCA. **(C, D)** KEGG pathway enrichment of ACOT7 in BRCA. **(E)** GSEA enrichment analysis.

To elucidate the potential mechanism of ACOT7 in BRCA, we systematically performed GO, KEGG, and GSEA enrichment analyses. GO analysis revealed that the expression of ACOT7 was significantly associated with epigenetic regulatory pathways such as miRNA-mediated gene silencing and centromeric chromatin assembly (**Figure 7A, B**). These results suggest that the function of ACOT7 in BRCA may not only be limited to lipid metabolism, but also be involved in tumorigenesis by regulating the epigenetic status of cells, thus revealing its potential role as a new regulatory factor. KEGG enrichment analysis indicated that ACOT7 may be a key node in the miRNA-mediated gene silencing network, and may regulate tumor progression by coordinating downstream target genes. Furthermore, ACOT7 was also linked to pathways including bitter taste transduction, the tumor immune microenvironment (neutrophil extracellular trap formation), and hormone metabolism (steroid hormone biosynthesis), providing clues for exploring its novel functions in BRCA immune regulation and hormonal responses (**Figure 7C, D**). GSEA enrichment analysis demonstrated that ACOT7 was significantly enriched in the oxidative phosphorylation pathway (**Figure 7E**), indicting that its high expression is positively correlated with a high-energy metabolic state in tumor cells. This suggests that ACOT7 may enhance mitochondrial function to promote energy production and biosynthesis, thereby supporting the rapid growth and invasion of breast cancer.

## 4 Discussion

The high heterogeneity of invasive breast carcinoma poses ongoing challenges for prognosis assessment and treatment strategy selection. The research frontier has shifted from a cancer-cell-centric paradigm to one that encompasses its surrounding immune context. Scientists have recognized the tumor immune microenvironment (TIME) as a dynamic nexus where persistent immune recognition and suppression collectively propel the trajectory of malignant progression, serving as an indispensable intrinsic driver of tumor advancement (8, 13, 14). Specifically, the presence of immunosuppressive characteristics, such as Treg cell enrichment and high PD-L1 expression, often predicts more rapid disease progression and poorer patient survival (15). Notably, metabolic reprogramming in tumor cells, especially disordered lipid metabolism, not only provides the energy foundation for their rapid proliferation but also drives the formation of an immunosuppressive milieu. Therefore, exploring the key molecules that drive disordered lipid metabolism and influence the tumor immune microenvironment is crucial to deepening our understanding of the progression mechanisms of invasive breast carcinoma.

ACOT7 is the most extensively studied major isoform of the ACOT7 family (16). It is highly and specifically expressed in tissues such as the brain, pituitary gland, bone marrow, and testis (12), and is associated with epilepsy (17) and Alzheimer’s disease (18). However, the observed upregulation of ACOT7 is highly expressed in various cancers, where it functions as a new proto-oncogene and potential therapeutic target, and an oncogenic factor. Lee S et al. found that colorectal cancer patients with high ACOT7 expression exhibit lower survival rates, and inhibition of ACOT7 significantly impairs cancer cell migration and invasion (19). Research by Jung SH and colleagues elucidated that ACOT7 exerts its function in breast and lung cancer by activating the PKCζ–p53–p21 pathway, thereby regulating the cell cycle (10). Nevertheless, the complete mechanistic role of ACOT7 in breast cancer remains to be further elucidated. In this study, by integrating GEO and TCGA databases and experimental validation, consistently revealed that the mRNA and protein expression levels of ACOT7 are significantly higher in BRCA tissues compared to normal tissues. This result was further confirmed in breast cell lines and clinical samples (RT-qPCR). The high expression pattern of ACOT7 suggests that it may play an important role in the pathogenesis of BRCA. Survival analysis based on the Kaplan-Meier plotter further indicated that high ACOT7 expression is significantly associated with shorter OS and DMFS in patients. These findings demonstrate that high ACOT7 expression is closely linked to poor prognosis in BRCA patients, positioning it as a potential valuable prognostic biomarker.

Accumulating evidence demonstrates that ACOT7 plays a critical role in the invasion and migration of various malignant tumors, including gastric cancer (20), non-small cell lung cancer (9, 21), hepatocellular carcinoma (22) and cutaneous melanoma (23). The specific molecular mechanisms of ACOT7 invasive ductal carcinoma of the breast remain unclear. To address this, our GO and KEGG enrichment analyses revealed that ACOT7 co-expression genes are primarily implicated in fatty acid metabolic processes and the PPAR signaling pathway, which is central to the modulation of cellular proliferation and apoptotic death. This result suggest that ACOT7 may hydrolyze acyl-CoA to alter intracellular levels of free fatty acids and lipid signaling molecules, thereby providing energy for rapid cancer cell proliferation and raw materials for biomembrane synthesis, while also participating in the regulation of key oncogenic pathways. Furthermore, GSEA analysis further indicated that high ACOT7 expression is positively correlated with inflammatory response, angiogenesis, and cell cycle pathways. Notably, we experimentally validated ACOT7 expression at both the tissue and cell levels. The results showed that in clinical samples, ACOT7 expression was significantly elevated in invasive carcinoma relative to adjacent non-cancerous tissues. In cell lines, ACOT7 expression was also significantly elevated in invasive cancer cells relative to normal mammary epithelial cells. These collective findings indicate that ACOT7 may function as a key oncogenic factor in IDC.

In recent years, the interaction between metabolic reprogramming and the tumor immune microenvironment (TIME) has emerged as a frontier in cancer research (24–26). Among these interactions, lipid metabolic reprogramming alters the production of bioactive lipids such as fatty acids (FAs). This not only supplies cancer cells with crucial components for membrane structures and fuel under stress conditions but also drives a tumor-promoting microenvironment through reshaping FAs metabolism (27, 28). TIME is composed of opposing immune cells and molecules populations. Its dynamic balance directly participates in regulating the tumor progression process and has a decisive impact on clinical outcomes (29, 30). It is noteworthy that the study by Li C et al. Demonstrated that ACOT7 directly drives resistance to vincristine (VCR) in retinoblastoma (RB) cells by regulating fatty acid metabolism and the autophagy process (31). This suggests that ACOT7-mediated lipid metabolic dysregulation may play a common role across different cancer types in malignant progression. Based on the above background, we utilized the TIMER database to interrogate the correlation between ACOT7 expression and immune cell infiltration in IDC. Further results indicated that at the level of immune cell infiltration, high ACOT7 expression showed a positive association with the presence of B cells and dendritic cells, alongside a concurrent reduction in the infiltration of macrophages, CD4^+^ T cells, and CD8^+^ T cells. This distinct immune cell infiltration pattern strongly suggests that ACOT7-mediated lipid metabolic reprogramming may contribute to shaping an immunosuppressive microenvironment - by functionally impairing key tumor-fighting immune cells such as cytotoxic T cells, thereby creating conditions conductive to tumor growth and immune escape. The results of this study, together with evidence of ACOT7 driven drug resistance in other cancers, provide a new perspective for a comprehensive understanding its carcinogenic mechanism: the function of ACOT7 extend beyond metabolic regulation within tumor cells to the remodeling of the entire tumor ecosystem.

In summary, this multi-dimensional study confirms that high ACOT7 expression is closely associated with poor prognosis in invasive ductal carcinoma of the breast, demonstrating its potential as a prognostic biomarker. Its mechanisms may involve driving lipid metabolic reprogramming, promoting the formation of an immunosuppressive microenvironment, and enhancing tumor cell invasion and migration capabilities. However, there are limitations to this study. Firstly, the specific downstream effector molecules through which ACOT7 regulates lipid metabolism via signaling pathways such as PPAR and influences the tumor immune microenvironment remain unclear and require further investigation. Secondly, this study did not validate ACOT7 expression at the protein level in IDC cell lines through experiments such as Western blot. Although bioinformatics analysis, mRNA detection in clinical samples, and IHC data from public databases collectively support the upregulation trend of ACOT7, the absence of direct quantitative protein expression data somewhat limits our understanding of its stability and post-translational regulation in IDC. Future research should integrate proteomic analyses to comprehensively confirm its expression profile and further elucidate its functional mechanisms. Finally, ACOT7 expression levels need to be validated in larger clinical cohorts, and its biological functions must be further confirmed through cellular experiments and animal models.

## 5 Conclusion

By integrating bioinformatics analysis with experimental validation, this study systematically elucidates the critical role of ACOT7 in the invasive ductal carcinoma (IDC) subtype of breast cancer. The results demonstrate that high ACOT7 expression serves as a prognostic marker for unfavorable outcomes, potentially through remodeling the tumor immune microenvironment and reprogramming metabolic pathways, thereby driving the malignant progression of IDC. These findings highlight the translational relevance of ACOT7, positioning it as both a prognostic indicator and a candidate for targeted intervention in IDC.

## Data Availability

The datasets presented in this study can be found in online repositories. The names of the repository/repositories and accession number(s) can be found in the article/supplementary material.
